# O03 Penicillin and cephalosporin cross-reactivity: a single-centre UK study

**DOI:** 10.1093/jacamr/dlad066.003

**Published:** 2023-06-14

**Authors:** Eve Duke, Zainab Abdelkader, Hannah Mooney, Fiona Byrne, Anoop Mistry, Sarah Denman

**Affiliations:** The Leeds Teaching Hospitals NHS Trust, Leeds, UK; The Leeds Teaching Hospitals NHS Trust, Leeds, UK; The Leeds Teaching Hospitals NHS Trust, Leeds, UK; The Leeds Teaching Hospitals NHS Trust, Leeds, UK; The Leeds Teaching Hospitals NHS Trust, Leeds, UK; The Leeds Teaching Hospitals NHS Trust, Leeds, UK

## Abstract

**Background:**

Penicillin allergy (PenA) labels limit antimicrobial options, and concerns about cross-reactivity between penicillin and cephalosporins can further restrict prescribing. Alternative therapies are associated with reduced efficacy, poorer safety profiles and increased healthcare costs. Emerging literature suggests the incidence of cross-reactivity between penicillins and cephalosporins may be lower than historically reported rates of 2–10%. Furthermore, there is a lack of real-time data evaluating the percentage of patients with PenA who tolerate a cephalosporin.

**Objectives:**

To determine the percentage of patients with a PenA label who tolerated cephalosporins in a secondary care setting.

**Methods:**

Patients prescribed a cephalosporin from 01 January 2016 to 31 December 2020 at Leeds Teaching Hospitals NHS Trust (LTHT) were identified using the inpatient electronic prescribing system. 1613 with a PenA label were extracted for analysis. The team reviewed and clarified whether each patient (i) had a PenA label that was a potential hypersensitivity reaction, intolerance, or unknown/undocumented label and (ii) tolerated a cephalosporin. Tolerance was defined as receiving ≥1 dose without adverse effects or subsequent cephalosporin allergy label. We recorded if allergy or intolerance was a documented reason for stopping the cephalosporin.

**Results:**

There were 1279 patients with a PenA label that received a cephalosporin. 555 (43%) had documentation in-keeping with a potential IgE-mediated allergic reaction to penicillins, 177 (14%) were deemed to have an intolerance and 547 (43%) had an unknown or undocumented label. 1263 (98.7%) patients tolerated a cephalosporin. Six (0.5%) patients stopped due to intolerance. One patient asked to stop therapy due to ‘changes’ in the skin. Nine (0.7%) patients developed possible allergic symptoms to cephalosporin; 4 patients developed a rash, 2 patients developed facial or lip swelling (without airway compromise), 1 patient developed hypotension, 1 patient developed difficulty breathing and 1 patient developed chest pain, breathlessness and itching. Of the 9 patients that had reactions; 5 had documented PenA labels that were classified as potential hypersensitivity reactions. Four patients had unknown or undocumented reactions to penicillins. Eight patients received a 2nd-generation cephalosporin (cefuroxime) and 1 received a 3rd generation (cefotaxime).

**Conclusions:**

The rate of potential hypersensitivity reaction following administration of a cephalosporin in patients with any PenA label at LTHT was 0.7%. The incidence of cephalosporin allergy is estimated to be 1–3% of the general population (with/without PenA label). We conclude that, for patients with a history of unverified, non-anaphylactic PenA label, any cephalosporin could be administered without an increased risk of hypersensitivity reaction.

